# 3,5-Dicarb­oxy-2,6-dimethyl­pyridinium chloride dihydrate

**DOI:** 10.1107/S1600536810017423

**Published:** 2010-05-15

**Authors:** Ji-Yuan Yao

**Affiliations:** a Ordered Matter Science Research Center, College of Chemistry and Chemical, Engineering, Southeast University, Nanjing 210096, People’s Republic of China

## Abstract

In the title compound, C_9_H_10_NO_4_^+^·Cl^−^·2H_2_O, both the cation and the anion have crystallographic twofold rotation symmetry; in the former, one N and one C atom lie on the rotation axis. In the crystal structure, the ions and water mol­ecules are linked *via* O—H⋯O, O—H⋯Cl and N—H⋯Cl hydrogen bonds into layers parallel to (101).

## Related literature

For the structure of a related 3,5-dicarb­oxy-2,6-dimethyl­pyridinium salt, see: Rowan & Holt (1997 ▶). For the ferroelectric properties of supra­molecular compounds, see: Ye *et al.* (2008 ▶); Hang *et al.* (2009 ▶). For a description of the Cambridge Structural Database, see: Allen *et al.* (2002 ▶).
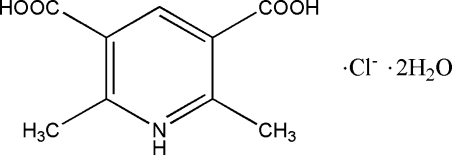


## Experimental

### 

#### Crystal data

C_9_H_10_NO_4_^+^·Cl^−^·2H_2_O
*M* *_r_* = 267.66
Monoclinic, 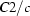

*a* = 8.2301 (10) Å
*b* = 10.7825 (10) Å
*c* = 13.882 (2) Å
β = 98.11 (3)°
*V* = 1219.5 (3) Å^3^
*Z* = 4
Mo *K*α radiation
μ = 0.33 mm^−1^
*T* = 293 K
0.50 × 0.50 × 0.50 mm

#### Data collection

Rigaku Mercury2 diffractometer
Absorption correction: multi-scan (*CrystalClear*; Rigaku, 2005 ▶) *T*_min_ = 0.938, *T*_max_ = 1.000
5826 measured reflections
1353 independent reflections
1204 reflections with *I* > 2σ(*I*)
*R*_int_ = 0.022

#### Refinement

*R*[*F* ^2^ > 2σ(*F* ^2^)] = 0.044
*wR*(*F* ^2^) = 0.120
*S* = 1.11
1353 reflections
91 parameters
H atoms treated by a mixture of independent and constrained refinement
Δρ_max_ = 0.26 e Å^−3^
Δρ_min_ = −0.21 e Å^−3^

### 

Data collection: *CrystalClear* (Rigaku, 2005 ▶); cell refinement: *CrystalClear*; data reduction: *CrystalClear* ; program(s) used to solve structure: *SHELXS97* (Sheldrick, 2008 ▶); program(s) used to refine structure: *SHELXL97* (Sheldrick, 2008 ▶); molecular graphics: *SHELXTL*/*PC* (Sheldrick, 2008 ▶); software used to prepare material for publication: *PRPKAPPA* (Ferguson, 1999 ▶).

## Supplementary Material

Crystal structure: contains datablocks I, global. DOI: 10.1107/S1600536810017423/rz2448sup1.cif

Structure factors: contains datablocks I. DOI: 10.1107/S1600536810017423/rz2448Isup2.hkl

Additional supplementary materials:  crystallographic information; 3D view; checkCIF report

## Figures and Tables

**Table 1:** Hydrogen-bond geometry (Å, °)

*D*—H⋯*A*	*D*—H	H⋯*A*	*D*⋯*A*	*D*—H⋯*A*
O1—H1⋯O1*W*^i^	0.82	1.72	2.537 (2)	173
N1—H1*A*⋯Cl1^ii^	0.95 (4)	2.21 (4)	3.160 (2)	180 (1)
O1*W*—H2⋯O2^iii^	0.79 (4)	1.95 (4)	2.720 (2)	166 (3)
O1*W*—H2*A*⋯Cl1^ii^	0.81 (4)	2.29 (4)	3.096 (2)	177 (3)

Symmetry codes: (i) 
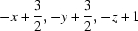
; (ii) 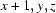; (iii) 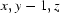.

**Fig. 1.**
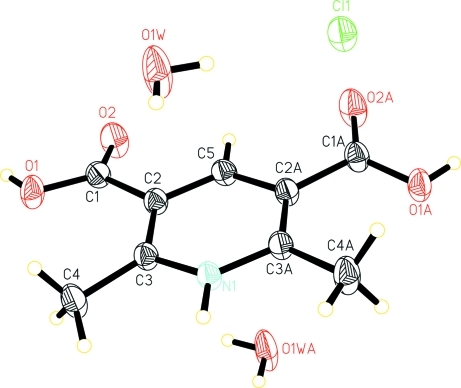
The molecular structure of the title compound, showing the atomic numbering scheme. Displacement ellipsoids are drawn at the 30% probability level. Atoms with suffix A are generated by the symmetry operation (2-x, y, 0.5-z).

**Fig. 2.**
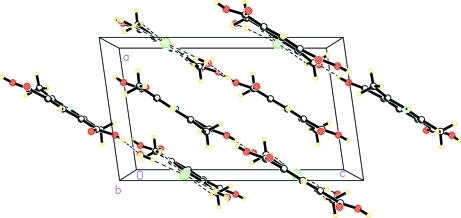
Crystal packing of the title compound viewed along the b axis. Dashed lines indicate hydrogen bonds.

## supplementary crystallographic information

### Comment

Organic and inorganic complexes or salts can develop supramolecular structures *via* multiple hydrogen-bonding systems by self-assembly of components which contain abundant hydrogen-bonding sites (Rowan & Holt, 1997). The present study is a part of systematic investigation of ferroelectric materials (Ye *et al.*, 2008; Hang *et al.*, 2009) that include metal-organic coordination compounds with organic ligands or compounds whose structures consist both of organic and inorganic building fragments.

The asymmetric unit of the title compound is composed of a half of a 3,5-dicarboxy-2,6-dimethylpyridinium cation, a half of a chloride anion and a water molecule. Both cation and anion have crystallographically imposed twofold rotation symmetry (Fig. 1). In the cation, the C—O bond lengths in the carboxylic group (C1—O1 = 1.300 (2) Å; C1—O2 = 1.218 (2) Å) conform to the expected values (Allen, 2002). The C3—N1—C3 angle of 126.6 (2) ° corresponds closely to the average value found in protonated pyridinium ions (122.0 (2) °). In the crystal structure (Fig. 2), the 3,5-dicarboxy-2,6-dimethylpyridinium cations, the chloride anions and the water molecules are linked *via* O—H···O, O—H···Cl and N—H···Cl hydrogen bonds (Table 1) to form two-dimensional layers parallel to the (101) plane. Dielectric studies (capacitance and dielectric loss measurements) were performed on powder samples of the title compound pressed into tablets on the surfaces of which a conducting carbon glue was deposited. The automatic impedance TongHui 2828 Analyzer has been used. In the measured temperature ranges (80 to 480 K, m.p. > 480 K), the structure showed no dielectric anomaly.

### Experimental

2,6-Dimethylpyridine-3,5-dicarboxylic acid (1.95 g , 10 mmol) and concentrated hydrochloric acid (10 mmol) were dissolved in methanol (25 ml). The solution was filtered and left at room temperature for 5 days. Colourless crystals suitable for X-ray analysis were obtained by slow evaporation of the solvent.

### Refinement

The pyridinium and water H atoms were located in a difference Fourier map and refined freely. All other H atoms were calculated geometrically and allowed to ride on their parent atoms, with C—H = 0.93-0.97 Å, O—H = 0.82 Å, and with *U*_iso_(H) = 1.5 *U*_eq_(C, O) or 1.2 *U*_eq_(C) for the aromatic H atom.

### Crystal data

**Table:**

C_9_H_10_NO_4_^+^·Cl^−^·2H_2_O	*F*(000) = 560
*M**_r_* = 267.66	*D*_x_ = 1.458 Mg m^−^^3^
Monoclinic, *C*2/*c*	Mo *K*α radiation, λ = 0.71073 Å
Hall symbol: -C 2yc	Cell parameters from 1418 reflections
*a* = 8.2301 (10) Å	θ = 3.3–27.1°
*b* = 10.7825 (10) Å	µ = 0.33 mm^−^^1^
*c* = 13.882 (2) Å	*T* = 293 K
β = 98.11 (3)°	Prism, colourless
*V* = 1219.5 (3) Å^3^	0.50 × 0.50 × 0.50 mm
*Z* = 4	

### Data collection

**Table:**

Rigaku Mercury2 diffractometer	1353 independent reflections
Radiation source: fine-focus sealed tube	1204 reflections with *I* > 2σ(*I*)
graphite	*R*_int_ = 0.022
Detector resolution: 13.6612 pixels mm^-1^	θ_max_ = 27.1°, θ_min_ = 3.1°
CCD_Profile_fitting scans	*h* = −10→10
Absorption correction: multi-scan (*CrystalClear*; Rigaku, 2005)	*k* = −13→13
*T*_min_ = 0.938, *T*_max_ = 1.000	*l* = −17→17
5826 measured reflections	

### Refinement

**Table:**

Refinement on *F*^2^	Primary atom site location: structure-invariant direct methods
Least-squares matrix: full	Secondary atom site location: difference Fourier map
*R*[*F*^2^ > 2σ(*F*^2^)] = 0.044	Hydrogen site location: inferred from neighbouring sites
*wR*(*F*^2^) = 0.120	H atoms treated by a mixture of independent and constrained refinement
*S* = 1.11	*w* = 1/[σ^2^(*F*_o_^2^) + (0.0618*P*)^2^ + 0.5934*P*] where *P* = (*F*_o_^2^ + 2*F*_c_^2^)/3
1353 reflections	(Δ/σ)_max_ < 0.001
91 parameters	Δρ_max_ = 0.26 e Å^−^^3^
0 restraints	Δρ_min_ = −0.21 e Å^−^^3^

### Special details

**Table:**

Geometry. All esds (except the esd in the dihedral angle between two l.s. planes) are estimated using the full covariance matrix. The cell esds are taken into account individually in the estimation of esds in distances, angles and torsion angles; correlations between esds in cell parameters are only used when they are defined by crystal symmetry. An approximate (isotropic) treatment of cell esds is used for estimating esds involving l.s. planes.
Refinement. Refinement of *F*^2^ against ALL reflections. The weighted *R*-factor wR and goodness of fit *S* are based on *F*^2^, conventional *R*-factors *R* are based on *F*, with *F* set to zero for negative *F*^2^. The threshold expression of *F*^2^ > σ(*F*^2^) is used only for calculating *R*-factors(gt) etc. and is not relevant to the choice of reflections for refinement. *R*-factors based on *F*^2^ are statistically about twice as large as those based on *F*, and *R*- factors based on ALL data will be even larger.

### Fractional atomic coordinates and isotropic or equivalent isotropic displacement parameters (Å^2^)

**Table:**

	*x*	*y*	*z*	*U*_iso_*/*U*_eq_	
Cl1	0.0000	0.47434 (6)	0.2500	0.0491 (2)	
O1	0.80009 (19)	0.97343 (12)	0.46240 (10)	0.0527 (4)	
H1	0.7609	1.0230	0.4976	0.079*	
N1	1.0000	0.76737 (19)	0.2500	0.0380 (4)	
O1W	0.8401 (3)	0.38706 (18)	0.42729 (16)	0.0882 (8)	
C1	0.8456 (2)	1.03196 (17)	0.38880 (13)	0.0440 (4)	
O2	0.8329 (2)	1.14346 (13)	0.37602 (13)	0.0691 (5)	
C2	0.9224 (2)	0.95307 (16)	0.31833 (12)	0.0388 (4)	
C3	0.9200 (2)	0.82364 (16)	0.31650 (12)	0.0387 (4)	
C4	0.8353 (3)	0.73948 (19)	0.37910 (16)	0.0569 (5)	
H4A	0.8362	0.6563	0.3545	0.085*	
H4B	0.7240	0.7664	0.3784	0.085*	
H4C	0.8915	0.7417	0.4446	0.085*	
C5	1.0000	1.0148 (2)	0.2500	0.0395 (5)	
H5	1.0000	1.1011	0.2500	0.047*	
H1A	1.0000	0.679 (4)	0.2500	0.066 (9)*	
H2	0.844 (4)	0.315 (4)	0.422 (2)	0.097 (11)*	
H2A	0.880 (4)	0.409 (3)	0.380 (3)	0.094 (10)*	

### Atomic displacement parameters (Å^2^)

**Table:**

	*U*^11^	*U*^22^	*U*^33^	*U*^12^	*U*^13^	*U*^23^
Cl1	0.0579 (4)	0.0409 (4)	0.0525 (4)	0.000	0.0217 (3)	0.000
O1	0.0749 (9)	0.0434 (8)	0.0461 (8)	0.0062 (6)	0.0306 (7)	−0.0030 (5)
N1	0.0476 (11)	0.0288 (10)	0.0412 (11)	0.000	0.0183 (8)	0.000
O1W	0.152 (2)	0.0408 (9)	0.0924 (14)	−0.0033 (10)	0.0889 (15)	−0.0088 (8)
C1	0.0525 (10)	0.0364 (9)	0.0466 (10)	−0.0028 (7)	0.0190 (8)	−0.0070 (7)
O2	0.1067 (13)	0.0324 (7)	0.0792 (11)	−0.0005 (7)	0.0505 (9)	−0.0073 (7)
C2	0.0440 (9)	0.0346 (8)	0.0403 (9)	−0.0001 (6)	0.0149 (7)	−0.0022 (6)
C3	0.0455 (9)	0.0335 (9)	0.0399 (9)	−0.0003 (6)	0.0160 (7)	−0.0009 (6)
C4	0.0809 (14)	0.0382 (10)	0.0607 (12)	−0.0052 (9)	0.0414 (11)	0.0003 (8)
C5	0.0453 (12)	0.0294 (11)	0.0459 (13)	0.000	0.0137 (10)	0.000

### Geometric parameters (Å, °)

**Table:**

O1—C1	1.300 (2)	C2—C5	1.386 (2)
O1—H1	0.8200	C2—C3	1.396 (3)
N1—C3	1.3513 (18)	C3—C4	1.495 (2)
N1—C3^i^	1.3513 (18)	C4—H4A	0.9600
N1—H1A	0.95 (4)	C4—H4B	0.9600
O1W—H2	0.79 (4)	C4—H4C	0.9600
O1W—H2A	0.81 (4)	C5—C2^i^	1.386 (2)
C1—O2	1.218 (2)	C5—H5	0.9300
C1—C2	1.501 (2)		
			
C1—O1—H1	109.5	N1—C3—C4	115.88 (16)
C3—N1—C3^i^	126.6 (2)	C2—C3—C4	127.09 (15)
C3—N1—H1A	116.68 (10)	C3—C4—H4A	109.5
C3^i^—N1—H1A	116.68 (11)	C3—C4—H4B	109.5
H2—O1W—H2A	101 (3)	H4A—C4—H4B	109.5
O2—C1—O1	124.43 (16)	C3—C4—H4C	109.5
O2—C1—C2	120.00 (16)	H4A—C4—H4C	109.5
O1—C1—C2	115.55 (16)	H4B—C4—H4C	109.5
C5—C2—C3	118.33 (15)	C2^i^—C5—C2	122.6 (2)
C5—C2—C1	116.78 (16)	C2^i^—C5—H5	118.7
C3—C2—C1	124.89 (15)	C2—C5—H5	118.7
N1—C3—C2	117.01 (15)		

Symmetry codes: (i) −*x*+2, *y*, −*z*+1/2.

### Hydrogen-bond geometry (Å, °)

**Table:**

*D*—H···*A*	*D*—H	H···*A*	*D*···*A*	*D*—H···*A*
O1—H1···O1W^ii^	0.82	1.72	2.537 (2)	173
N1—H1A···Cl1^iii^	0.95 (4)	2.21 (4)	3.160 (2)	180 (1)
O1W—H2···O2^iv^	0.79 (4)	1.95 (4)	2.720 (2)	166 (3)
O1W—H2A···Cl1^iii^	0.81 (4)	2.29 (4)	3.096 (2)	177 (3)

Symmetry codes: (ii) −*x*+3/2, −*y*+3/2, −*z*+1; (iii) *x*+1, *y*, *z*; (iv) *x*, *y*−1, *z*.
